# Romantic Relationship Satisfaction and Parent-Infant Bonding During the Transition to Parenthood: An Attachment-Based Perspective

**DOI:** 10.3389/fpsyg.2020.02068

**Published:** 2020-08-28

**Authors:** Kathleen K. Little, Laura E. Sockol

**Affiliations:** ^1^ Department of Psychology, Davidson College, Davidson, NC, United States; ^2^ Department of Psychology, University of Miami, Miami, FL, United States

**Keywords:** adult attachment, parental divorce, parent-infant bonding, postpartum, relationship satisfaction

## Abstract

An important element of well-being during the transition to parenthood is new parents’ relationships with their partners and babies. Attachment theory posits that early caregiving experiences influence close relationships throughout the lifespan. Disruptions to the parent-child relationship, such as parental divorce or separation, may therefore have intergenerational effects as adult children of divorce navigate changes in their later relationships. This study examined whether new parents who have experienced a divorce or separation in their family of origin report greater romantic relationship dissatisfaction or impairment in the parent-infant bond during the early postpartum period, and if these associations are mediated by adult attachment. First-time parents of infants through 6 months of age (*N* = 94) completed measures of adult attachment, romantic relationship satisfaction, and parent-infant bonding. New parents who had experienced parental divorce or separation did not differ from those from intact families with regard to romantic relationship satisfaction, parent-infant bonding, attachment anxiety, or attachment avoidance. Attachment anxiety and avoidance were both associated with romantic relationship dissatisfaction and greater impairment in the parent-infant bond. These findings suggest that the experience of parental divorce or separation, in and of itself, does not confer increased risk for negative relational outcomes among new parents. Securely attached adults, regardless of their own parents’ marital status, report more positive relationships with their partners and infants during the early postpartum period.

## Introduction

The transition to parenthood can affect well-being in a range of domains. Most research on distress in perinatal populations has focused on psychological disorders, such as depression (Cameron et al., 2016; Woody et al., 2017), anxiety disorders (Leach et al., 2016; Dennis et al., 2017), obsessive-compulsive disorder (Frías et al., 2014), and post-traumatic stress disorder (Grekin and O’Hara, 2014). Another important element of parental well-being is the quality of new parents’ relationships with their partners and babies. In this study, we apply an attachment theory perspective to investigate whether the experience of parental divorce or separation is associated with romantic relationship satisfaction and parent-infant bonding among first-time parents during the early postpartum period.

## Relationships During the Transition to Parenthood

Individuals and couples navigate many potential challenges when they become parents, including sleep disruption, introduction of novel responsibilities, re-negotiation of the division of household labor, and changes in the amount and quality of time that couples spend together (Houlston et al., 2013). Many couples experience a small but significant decline in relationship satisfaction during the transition to parenthood (Mitnick et al., 2009). This can have important consequences; for example, postpartum relationship dissatisfaction is associated with increased risk for relationship dissolution during the first 3 years of parenthood (Røsand et al., 2014). Relationship dissatisfaction during the perinatal period is also associated with increased risk for depression and anxiety for both men and women (Pilkington et al., 2015).

New parents may also experience difficulties in their relationships with their babies. *Parent-infant bonding* refers to parent’s affective responses and cognitive evaluations of their relationship with their infant (Kinsey and Hupcey, 2013). It is important to distinguish parent-infant bonding, which represents the parent’s perception of this relationship, from *infant attachment*, which represents the quality of the infant’s relationships with their caregivers. In community samples, the prevalence of clinically significant disturbances to the parent-infant bond approaches 10% (Skovgaard et al., 2007); among mothers referred for perinatal services, the prevalence of severe disruption to the mother-infant bond exceeds 25% (Brockington, 2011). Impairments in parent-infant bonding are associated with less sensitive and appropriate parenting (Noorlander et al., 2008; Dayton et al., 2010; Hall et al., 2014), later disruptions to the parent-child relationship (de Cock et al., 2016), and increased risk for child cognitive and behavioral difficulties (Hairston et al., 2011; de Cock et al., 2017). Most research on parent-infant bonding has been conducted with mothers, but there is also clear evidence for the importance of the father-infant bond (Coleman et al., 2004).

Although some new parents develop problems in their relationships with their partners or infants, this experience is far from universal. There is a wide variability in the nature and degree of changes in romantic relationship satisfaction across the transition to parenthood, with approximately half of couples reporting stable or even improved relationship quality (Kluwer, 2010). Similarly, most new parents do not experience clinically significant impairments to the parent-infant bond, even in the context of stressors, such as maternal depression (Brockington et al., 2006). Given the variability in new parents’ experiences of relational difficulties during the transition to parenthood, it is important to identify processes that contribute to both risk and resilience as parents, couples, and families navigate the challenges associated with the early months of parenthood.

## Attachment Theory

Attachment theory provides a valuable lens through which to understand new parents’ relationships with their partners and infants (for an overview of attachment theory, see Thompson, 2013). According to this theory, patterns of behavior and expectations that develop through early interactions with caregivers are often imposed on new relationships later in life (Bowlby, 1988). Early experiences with caregivers lead children to form representations, or *inner working models*, of the self and others, which can be used to characterize an individual’s *attachment style* (Marvin et al., 2016). Securely attached individuals are confident that others will be available and responsive to their needs, while insecurely attached individuals may be uncertain of others’ availability or responsiveness or may not expect to receive support from others (Bowlby, 1988). These foundational concepts in attachment theory were later extended to adult relationships, including romantic relationships (Hazan and Shaver, 1987; Shaver and Mikulincer, 2009). Adult attachment is often characterized along two dimensions: *attachment anxiety*, which is characterized by uncertainty that one’s feelings are reciprocated and concerned that a partner will leave, and *attachment avoidance*, which is characterized by difficulty with trust and intimacy (Bartholomew and Horowitz, 1991).

## Attachment Security and Relationship Outcomes

There is clear evidence that attachment style is associated with the quality of important relationships in adulthood. Meta-analyses find that, among adults, both attachment anxiety and avoidance are associated with romantic relationship dissatisfaction (Li and Chan, 2012; Hadden et al., 2014; Candel and Turliuc, 2019). Attachment style may play a particularly important role in adaptation during periods of stress or change, including the transition to parenthood (Mikulincer and Florian, 1998). Among new parents, securely attached individuals report greater romantic relationship satisfaction; their partners also evaluate these relationships more positively (Hirschberger et al., 2009).

Adult attachment style is also related to new parents’ relationships with their infants. Securely attached mothers report more positive mother-infant bonding during the first year postpartum (van Bussel et al., 2010; Wilkinson and Mulcahy, 2010). In contrast, insecure maternal attachment is associated with a range of impairments to the mother-infant bond. Mothers whose attachment styles are characterized by anxiety and ambivalence report greater anxiety in their relationships with their infants, while mothers with avoidant attachment styles report greater rejection and anger (Hairston et al., 2018). Mothers with disorganized attachment styles, characterized by high levels of both anxiety and avoidance, also report more impaired bonding (Nonnenmacher et al., 2016). To our knowledge, no previous studies have directly assessed the relationship between paternal attachment and the father-infant bond.

## Effects of Parental Divorce/Separation on Attachment Security and Relationship Outcomes

The experience of parental divorce or separation in an individual’s family of origin may affect their interactions with caregivers in ways that increase risk for insecure attachment. For example, children of divorced parents may experience reduced contact with one or both caregivers due to custody arrangements, and the quality of interactions with caregivers may be affected by increased stress or new responsibilities (Feeney and Monin, 2016; Nielsen, 2018). There is evidence that parental divorce is associated with insecure attachment among children (Clarke-Stewart et al., 2000), adolescents (Ozen, 2004), and adults (Crowell et al., 2009; Fraley and Heffernan, 2013). Furthermore, among young children classified as securely attached, the experience of a subsequent parental divorce is associated with greater risk for insecure attachment in adulthood (Waters et al., 2000). The association between parental divorce/separation and adult attachment insecurity has also been demonstrated during the transition to parenthood; expectant parents whose own parents divorced or separated are more likely to be classified as insecurely attached than those from intact families of origin (Riggs and Jacobvitz, 2002).

This increased risk for attachment insecurity suggests that the experience of parental divorce or separation may affect relationships throughout the lifespan. There is evidence that adults from intact families of origin experience more positive relationship outcomes in adulthood. An early meta-analysis found a small but significant increase in the prevalence of divorce/separation among adults whose own parents had divorced or separated (Amato and Keith, 1991). Adults whose parents divorced or separated also report greater dissatisfaction in their romantic relationships (Amato and Keith, 1991; Mustonen et al., 2011). Notably, no studies have directly assessed whether new parents’ experiences of divorce or separation in their own families of origin are associated with parent-infant bonding.

Although a history of parental divorce or separation appears to increase risk for negative relational outcomes, many adult children of divorced or separated parents do not experience these difficulties. In fact, some report relational benefits resulting from parental divorce, including closer relationships with siblings and other family members and expansion of social networks (Halligan et al., 2014; Roper et al., 2020). There is also evidence that the association between parental divorce and negative outcomes may be decreasing as divorce has become more common and less stigmatized (Auersperg et al., 2019). These findings suggest a need for further research on the association between parental divorce and relational outcomes in adulthood, especially during periods of increased stress, such as the transition to parenthood.

## The Current Study

In this study, we investigated whether new parents with a history of parental divorce or separation experience greater romantic relationship dissatisfaction or impairments in the parent-infant bond during the early postpartum period, and whether these associations are mediated by adult attachment style. Consistent with previous research, we predicted that first-time parents whose parents had divorced or separated would report lower romantic relationship satisfaction and greater impairments in the parent-infant bond. We further predicted that these parents would report higher levels of attachment anxiety and avoidance. Finally, we hypothesized that associations between parental divorce/separation and relationship outcomes would be mediated by adult attachment.

## Method

Individuals were eligible to participate if they were a first-time parent of a child aged 6 months or younger, between the ages of 18 and 45, resided in the United States, and were currently in a relationship. Participants were recruited online and completed all study materials at a single time-point through a secure online interface. After providing informed consent and confirming eligibility, participants completed measures of adult attachment style, romantic relationship satisfaction, and parent-infant bonding (described below) in a random order. Participants then provided information about their family of origin, including a retrospective report of their parents’ relationship status and demographic information. The study received approval from the Institutional Review Board of Davidson College prior to data collection.

### Measures

#### Adult Attachment Style

Attachment was assessed with the Experiences in Close Relationships Scale (ECR; Brennan et al., 1998), a 36-item measure characterizing general feelings in romantic relationships that includes subscales for anxious (e.g., “I need a lot of reassurance that I am loved by my partner”) and avoidant (e.g., “I find it difficult to allow myself to depend on romantic partners”) elements of attachment. Scores on each subscale range from 18 to 126, with higher scores indicating greater attachment insecurity. Internal reliability was excellent for both anxiety (ECR-ANX, *α* = 0.92) and avoidance (ECR-AVO, *α* = 0.93).

#### Romantic Relationship Satisfaction

Relationship satisfaction was assessed with the Dyadic Adjustment Scale (DAS; Spanier, 1976), a 32-item self-report measure that provides an overall indicator of romantic relationship quality. Scores on the DAS range from 0 to 151, with higher scores indicating greater relationship satisfaction. Internal reliability of the DAS was excellent (*α* = 0.93).

#### Parent-Infant Bonding

Parent-infant bonding was assessed with the Parental Bonding Questionnaire (PBQ; Brockington et al., 2001). The original PBQ includes 25 items assessing four domains of the parent-infant relationship: impaired bonding, rejection/anger, anxiety, and risk of abuse (Brockington et al., 2001). As previous studies have found that the risk of abuse subscale has low sensitivity and reliability (e.g., Brockington et al., 2006), these items were excluded from the current study. Scores on our revised 23-item version of the PBQ range from 0 to 115, with higher scores indicating greater impairment of the parent-infant bond. Internal reliability for the PBQ was excellent (*α* = 0.91).

#### Family of Origin and Demographic Characteristics

Participants were first asked if both parents were living; participants who did not report loss of a parent were asked to indicate the current status of their parents’ relationship to one another. Participants who reported that their parents were divorced or separated also provided their age at the time of the divorce/separation.

Following the completion of study materials, participants provided the following demographic information: age, gender, relationship status, race, ethnicity, level of education, employment status, child age, and child gender.

### Participants

Of the 142 eligible participants who initiated the study, 116 (82%) completed all study measures and reported information regarding their parents’ relationship status. As parental loss may also influence adult attachment (Brennan and Shaver, 1998), 22 participants who had experienced the loss of a parent were excluded from analyses, resulting in a final sample of 94 participants.

Demographic characteristics of the sample are presented in Table 1. Most participants were women and were predominantly white, non-Hispanic/Latinx, and married. The majority had completed a 4-year college degree and were currently employed. Average child age was between 3 and 4 months, with comparable proportions of male and female children.

**Table 1:** Sample demographic characteristics (*N* = 94).

Characteristic	Range	*M*(*SD*)	Median
Age (Years)	20–45	30.6(4.6)	31.0
Child age (Weeks)	1–28	14.4(6.6)	14.0
Age at parental divorce/separation (*n* = 27)	1–31	12.3(8.1)	9.5
	*n*	%	
Gender
Man	12	13	
Woman	81	86	
Non-binary	2	2	
Race
White/Caucasian	80	85	
Black/African-American	7	7	
Asian/Asian-American	6	6	
Native American/Alaska Native	2	2	
Other	3	3	
Ethnicity
Non-Hispanic/Latinx	83	88	
Hispanic/Latinx	11	12	
Education
High school diploma/GED	6	6	
Some college	14	15	
Associate degree/Trade school	7	7	
Bachelor’s degree	23	24	
Graduate/professional school	44	47	
Employment status
Employed, full-time	68	72	
Employed, part-time	6	6	
Employed, other	3	3	
Not employed	17	18	
Relationship status
Married	78	83	
In a relationship, living together	12	13	
In a relationship, not living together	4	4	
Child gender
Boy	48	51	
Girl	46	49	
Parental divorce status
Married	65	69	
In a committed relationship, not married	2	2	
Separated	6	6	
Divorced	21	22	

As participants could endorse more than one option for gender and racial identity, percentages in these categories may sum to greater than 100. One participant classified their gender as a non-binary woman. Three participants endorsed more than one racial identity category: two participants endorsed two categories (White and Native American/Alaska Native and Black/African-American and Other), and one participant endorsed three categories (White/Caucasian, Black/African-American, and Native American/Alaska Native).

## Results

### Descriptive Statistics

Descriptive statistics for the primary study measures are presented in Table 2. As scores on the DAS and PBQ were skewed, these variables were winsorized prior to analyses; outliers ≥3.29 standard deviations from the mean were replaced with the value ±3.29 standard deviations from the mean (Field, 2013). We first assessed whether demographic characteristics were associated with study outcomes. After correcting for multiple comparisons, we found no significant associations between any demographic characteristic and study outcomes and did not include these variables in subsequent analyses.

**Table 2:** Descriptive statistics and intercorrelations among study measures.

	*M*(*SD*)	Range	Attachment anxiety	Attachment avoidance	Romantic relationship satisfaction	Parent-infant bonding
Attachment anxiety (ECR-ANX)	60.95(21.14)	20–104	(0.92)			
Attachment avoidance (ECR-AVO)	40.90(18.01)	18–102	0.48^***^	(0.93)		
Romantic relationship satisfaction (DAS)	116.83(16.44)	38–148	−0.31^**^	−0.63^***^	(0.93)	
Parent-infant bonding (PBQ)	16.88(11.52)	0–72	0.27^**^	0.29^**^	−0.19^†^	(0.91)

Descriptive statistics are provided for un-transformed data. Internal reliability coefficients (Cronbach’s alpha) are presented in parentheses on the diagonal.

† *p* < 0.10;

** *p* < 0.01;

*** *p* < 0.001.

The pattern of correlations among the study measures was consistent with our hypotheses (see Table 2). Attachment anxiety and avoidance were moderately correlated with one another. There was a marginally significant association between romantic relationship satisfaction and parent-infant bonding. Attachment anxiety and avoidance were both significantly associated with romantic relationship dissatisfaction and impairments to the parent-infant bond.

Most participants’ parents were married (69%) or in a committed relationship with one another (2%); nearly one-third of the sample reported that their parents were divorced (22%) or separated (6%). Among participants whose parents were divorced or separated, the age at parental divorce/separation ranged from 1 to 31 years, with a mean of 12.3 years (*SD* = 8.1, median = 9.5).

### Associations Between Parental Divorce/Separation and Relationship Outcomes

We first compared the two groups of participants on adult attachment, romantic relationship satisfaction, and parent-infant bonding using a series of independent-samples *t*-tests (see Table 3). There were no significant differences in any of these outcomes between participants whose parents were divorced or separated compared to participants from intact families of origin (all values of *p* > 0.10).

**Table 3:** Differences in study outcomes between participants from intact families (*n* = 67) and participants with divorced/separated parents (*n* = 27).

	Intact family of origin *M*(*SD*)	Parents divorced/separated *M*(*SD*)	*p*	*d*
Attachment anxiety (ECR-ANX)	59.91(19.95)	63.52(24.06)	0.457	0.16
Attachment avoidance (ECR-AVO)	39.42(18.18)	44.59(17.36)	0.209	0.29
Romantic relationship satisfaction (DAS)	116.92(14.98)	117.52(16.43)	0.865	0.04
Parent-infant bonding (PBQ)	17.33(11.20)	15.15(9.55)	0.377	−0.21

Although we did not find differences in romantic relationship satisfaction or parent-infant bonding between the two groups of participants, it is possible to observe significant indirect effects even when the corresponding direct effect is not significant (Hayes, 2018). To assess this, we conducted a series of mediation analyses investigating whether the relationships between parental divorce status and relationship outcomes were mediated by adult attachment. Attachment anxiety and avoidance were assessed as potential mediators of the relationships between parental divorce status and romantic relationship satisfaction and parent-infant bonding in separate models. Mediation analyses were conducted using version 3.4 of Hayes (2019) PROCESS macro for SPSS, using 10,000 bootstrap samples.

**Figure 1:**
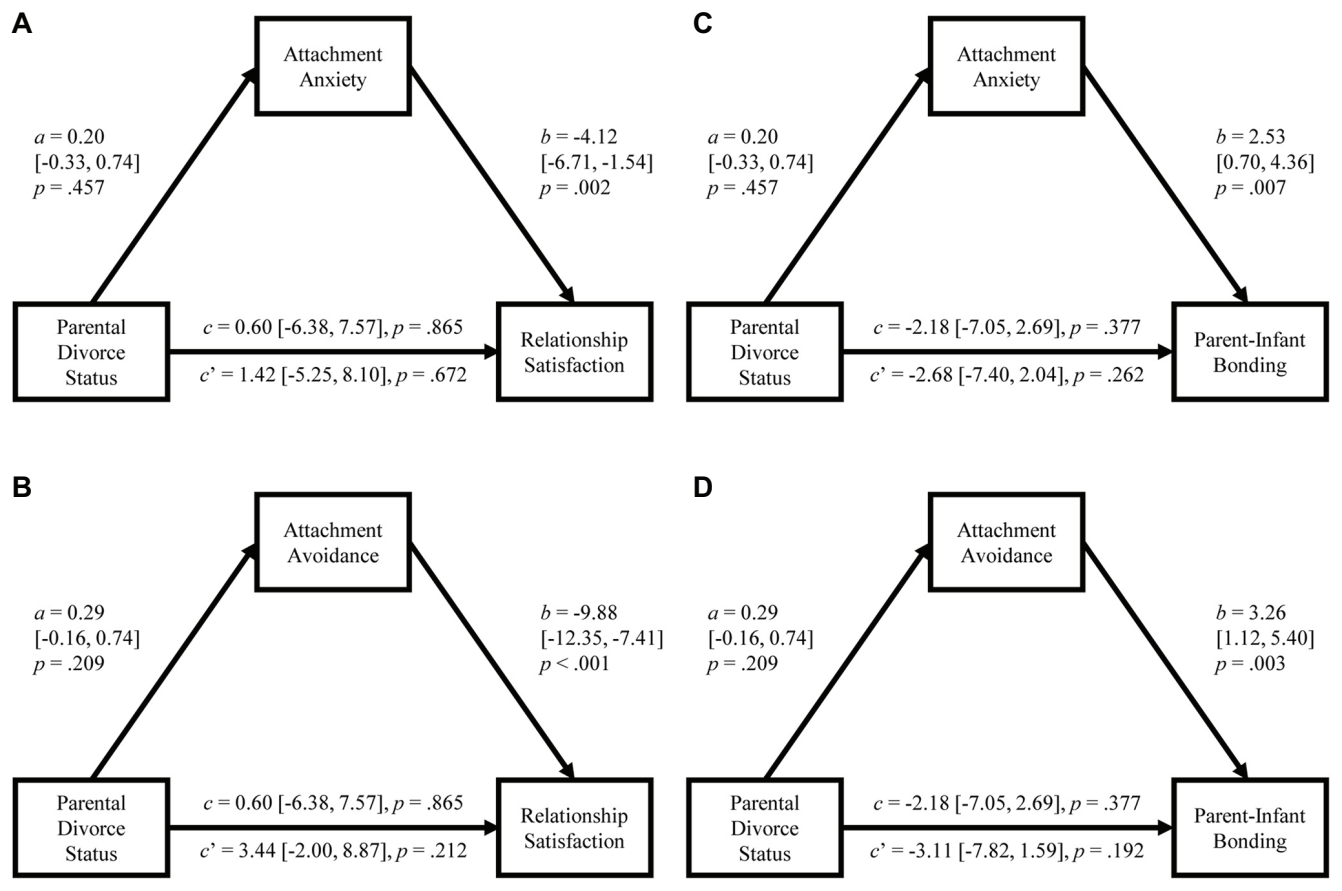
Results of mediation analyses. Separate analyses were conducted assessing attachment anxiety (A,C) and attachment avoidance (B,D) as potential mediators of the association between parental divorce status and romantic relationship satisfaction (A,B) and parent-infant bonding (B,D). Path a represents the effect of parental divorce status (0 = not divorced/separated, 1 = divorced/separated) on attachment, path b represents the direct effect of attachment on the relationship outcome (controlling for parental divorce status), path *c* represents the total effect of parental divorce status on the relationship outcome, and path *c*’ represents the direct effect of parental divorce status on the relationship outcome (controlling for attachment). All coefficients are presented as unstandardized estimates. To facilitate interpretability of the unstandardized coefficients, participants’ average scores on the ECR subscales were used for the adult attachment variables.

Results of the mediation analyses indicated that parental divorce status was not significantly associated with either attachment anxiety or avoidance (see Figure 1). Attachment anxiety and avoidance were both significantly associated with lower romantic relationship satisfaction (see Figures 1A,B). The 95% bias-corrected bootstrap CIs for the indirect effects of attachment on romantic relationship satisfaction ranged from −3.83 to 1.46 for anxiety and −7.57 to 1.38 for avoidance, indicating that there was not a significant indirect effect of parental divorce status on romantic relationship satisfaction *via* either attachment anxiety or avoidance. There was also not a significant direct effect of parental divorce status on romantic relationship satisfaction after controlling for attachment anxiety or avoidance.

Results of mediation analyses revealed a comparable pattern for parent-infant bonding (see Figures 1C,D). Both attachment anxiety and avoidance were significantly associated with greater impairments in parent-infant bonding. The 95% bias-corrected bootstrap CIs for the indirect effects of attachment ranged from −0.97 to 2.32 for anxiety and −0.47 to 2.96 for avoidance, indicating that there was not a significant indirect effect of parental divorce status on parent-infant bonding *via* attachment anxiety or avoidance. There was also not a significant direct effect of parental divorce status on parent-infant bonding after controlling for attachment anxiety or avoidance.

## Discussion

Findings from this study did not support our hypotheses that a history of parental divorce or separation would be associated with insecure attachment, romantic relationship dissatisfaction, and impaired parent-infant bonding during the transition to parenthood. We did, however, find that adult attachment security is associated with both romantic relationship satisfaction and parent-infant bonding during the early postpartum period.

These findings provide a valuable counterpoint to prevailing trends in the study of the consequences of parental divorce and new parents’ romantic relationships, both of which typically emphasize potential negative outcomes (for reviews, see Mitnick et al., 2009; Feeney and Monin, 2016). In contrast, our findings suggest that many individuals who experience parental divorce or separation in their families of origin do not experience greater attachment insecurity or more problems in their relationships with their partners or children. To our knowledge, this is the first study to directly assess whether divorce or separation in a new parent’s family of origin is associated with impairments in the parent-infant bond. We found no evidence that these parents experience more difficulties in their early relationships with their babies.

These results are initially surprising, given that most studies find that parental divorce is associated with a greater risk for negative relational outcomes in adulthood (Feeney and Monin, 2016). One possible explanation is that parental divorce or separation may predict whether an individual pursues or achieves specific relational outcomes, such as choosing to marry or start a family, but may be less predictive of the quality of later relationships. As our sample was limited to new parents who were currently in a relationship, our findings may not be representative of single adults or non-parents. Consistent with this explanation, a similar pattern of results was observed in a longitudinal study of newlywed couples. Among these adults, who had established a successful relationship and made the decision to marry their partner, those whose own parents had divorced or separated had no greater risk of divorce during the first 6 years of marriage than newlyweds from intact families (Crowell et al., 2009).

Our findings provide clear evidence that insecure attachment is associated with relationship difficulties during the transition to parenthood. New parents with high levels of anxious and avoidant attachment were less satisfied in their romantic relationships; this finding is consistent with a large body of literature (Li and Chan, 2012; Hadden et al., 2014; Candel and Turliuc, 2019), as well as with theoretical models that emphasize the importance of attachment style during periods of transition (e.g., Mikulincer and Florian, 1998). We also found that new parents who reported high levels of anxious and avoidant attachment experienced greater impairments in the parent-infant bond. Importantly, we demonstrated that this association is present in a sample including both mothers and fathers. Previous studies have found that securely attached mothers report more positive mother-infant bonding (van Bussel et al., 2010; Wilkinson and Mulcahy, 2010), and that insecurely attached mothers are at greater risk for impairments to the mother-infant bond (Nonnenmacher et al., 2016; Hairston et al., 2018). Our findings extend this literature and provide evidence of increased risk for impaired father-infant bonding in the context of insecure paternal attachment.

Together, these findings suggest that individuals and couples may benefit from interventions addressing attachment security during the transition to parenthood. Research has identified characteristics of relationships that appear to be protective against declining relationship satisfaction among new parents, including the quality of interactions, engagement, and congruence of attitudes related to gender roles and the division of household labor (Houlston et al., 2013). Secure attachment might enhance these factors through skills that promote adaptive processes in couple interactions, including the ability to give and receive emotional support, conflict management skills, and affective regulation abilities (Sutton, 2019).

## Limitations and Future Directions

A significant limitation of the current study is our relatively small sample. Our analyses, particularly those assessing mediation, were underpowered; when associations between individual variables are small, mediation analyses may require samples of 400 or more participants (Fritz and MacKinnon, 2007). Although underpowered, the effect sizes we observed suggest small or negligible differences between new parents from intact families compared to those whose parents divorced or separated. In fact, participants from intact families reported slightly *more* impaired bonding than those whose parents had divorced or separated. Despite our small sample, this pattern of findings suggests that the absence of a relationship between parental divorce/separation and relational outcomes in this study is unlikely to be accounted for by inadequate statistical power.

A related limitation is that our sample included a small number of participants who had experienced parental divorce or separation; thus, we were unable to evaluate characteristics of the divorce/separation as predictors of attachment and relational outcomes. One important characteristic that should be addressed in future research is the age at which the parental divorce/separation occurred. In our sample, participants’ age at the time of divorce/separation ranged from 1 to 31 years. In an early study of the “intergenerational transmission of divorce,” Amato (1996) found that the relationship between parental divorce and marital status in adulthood was strongest when the divorce occurred when the child was 12 years old or younger. Similarly, parental divorce/separation appears to be more strongly associated with insecure attachment when it occurs earlier in childhood (Crowell et al., 2009; Fraley and Heffernan, 2013). We conducted exploratory analyses including only participants who were younger than 13 at the time of their parents’ divorce/separation (*n* = 15); although our findings remained stable, the very small sample of participants who experienced a parental divorce or separation prior to adolescence remains a concern. We were also unable to assess other important characteristics of the divorce/separation, such as the presence of parental conflict or changes in the amount or quality of contact with caregivers that are associated with long-term outcomes (Feeney and Monin, 2016). Future research with larger samples would allow for more robust evaluation of specific characteristics of parental divorce/separation that might influence attachment security and later relationships.

In addition to evaluating larger samples, future studies investigating associations among parental divorce/separation, attachment, and relational outcomes should include more diverse and representative samples. Our sample was primarily comprised of white, non-Hispanic/Latinx women. Although we found no differences in adult attachment, romantic relationship satisfaction, or parent-infant bonding related to participants’ demographic characteristics, our ability to detect potential differences was limited by inadequate representation of participants from specific demographic groups. While exploratory analyses found no evidence that gender moderated the relationships among parental divorce/separation, attachment, and relationship outcomes in our sample, other studies suggest that parental divorce/separation may be a stronger risk factor for negative relational outcomes for women (Crowell et al., 2009; Mustonen et al., 2011). Similarly, there is evidence that associations between early parent-child relationships and later adult attachment may vary across racial and ethnic groups (Lopez et al., 2000). Greater representation of participants from a wide range of demographic groups would allow for direct evaluation of whether associations between parental divorce/separation and relational outcomes vary according to parental characteristics, such as gender, sexual orientation, race/ethnicity, or socioeconomic status.

Finally, the present study was limited by its cross-sectional design and retrospective assessment of parental relationship status. Future research using longitudinal designs would allow for the prospective evaluation of the effects of parental divorce or separation on relational outcomes. In addition to providing an opportunity to assess the effects of parental divorce or separation on romantic relationship satisfaction and the parent-infant bond, such studies would also provide opportunities to evaluate whether adults whose parents are divorced or separated are less likely to pursue specific kinds of relationships in adulthood, or to become parents.

## Conclusion

Overall, these findings provide evidence that adult attachment is strongly associated with the quality of new parents’ relationships with their partners and babies during the early postpartum period. In contrast to previous research, we did not find that attachment security or relational outcomes were associated with the experience of divorce or separation in new parents’ families of origin. These findings highlight the importance of adult attachment and suggest that secure attachment may promote positive relational outcomes for parents, couples, and families during the transition to parenthood.

## Data Availability Statement

The datasets generated for this study are available on request to the corresponding author.

## Ethics Statement

The studies involving human participants were reviewed and approved by Davidson College Institutional Review Board. The patients/participants provided their written informed consent to participate in this study.

## Author Contributions

KL conceived the study. KL and LS designed the study. KL carried out data collection under the supervision of LS. KL and LS analyzed the data and wrote the manuscript. All authors read and approved the submitted version of the manuscript.

### Conflict of Interest

The authors declare that the research was conducted in the absence of any commercial or financial relationships that could be construed as a potential conflict of interest.
